# Cognitive Frailty And Risk Of Functional Disability In Older Japanese Adults: A 4-Year Prospective Study

**DOI:** 10.1093/geroni/igab046.2994

**Published:** 2021-12-17

**Authors:** Sanmei Chen, Takanori Honda, Tao Chen, Hiro Kishimoto, Shuzo Kumagai, Kenji Narazaki

**Affiliations:** 1 Graduate School of Biomedical and Health Sciences, Hiroshima University, Hiroshima, Hiroshima, Japan; 2 Kyushu University, Fukuoka, Fukuoka, Japan; 3 Tongji University, Department of Physical Education, Tongji University, Shanghai, China (People's Republic); 4 Kyushu University, Kyushu University, Fukuoka, Japan; 5 Kumagai Institute of Health Policy, Kasuga City, Fukuoka, Japan; 6 Fukuoka Institute of Technology, Fukuoka, Fukuoka, Japan

## Abstract

Background: Cognitive frailty is a newly proposed clinical entity, referring to concurrent cognitive impairment and physical frailty in the absence of dementia. The clinical significance of cognitive frailty remains poorly understood. We aimed to investigate the association between cognitive frailty and functional disability in older adults. Methods: A total of 1,644 non-demented older adults aged ≥65 years (mean age: 73 ± 6 years; men: 41.8%) and without functional disability at baseline were followed-up for 4 years. Cognitive frailty was defined as the presence of both physical frailty (based on the modified Cardiovascular Health Study criteria) and cognitive impairment (Mini-Mental State Examination score of <24 points). Functional disability was identified using the database of Japan’s Long-term Care Insurance System. Association between cognitive frailty and functional disability was assessed by using the Cox proportional hazard models. Results: During the follow-up, 152 participants were identified as being functionally disabled. There was a significant interaction between physical frailty and cognitive impairment on the development of functional disability (P <0.1). Compared with being robust both physically and cognitively, the hazard ratio (95% confidence interval) of functional disability was 8.40 (4.05-17.42) for cognitively frailty, after adjustment for age, sex, education, living alone, smoking, drinking, number of comorbidities (hypertension, stroke, chronic heart disease, diabetes, chronic kidney disease, poor hearing, poor vision, osteoarthritis or rheumatism, minor trauma fracture, or cancer). Conclusion: Cognitive frailty was associated with an increased risk of functional disability in community-dwelling older adults. Cognitive frailty could be an underrecognized risk factor for functional disability.

